# Efficacy of Human Amniotic Membrane Stem Cell-Conditioned Medium on Improving In Vitro Maturation Efficiency of Mouse Oocytes

**DOI:** 10.3390/cells15161454

**Published:** 2026-08-13

**Authors:** Kihae Ra, Eun Young Kim, Sung Keun Kang, Geon A Kim, Jeong Chan Ra

**Affiliations:** 1 College of Veterinary Medicine and Research Institute for Veterinary Science, Seoul National University, Seoul 08826, Republic of Korea; 2 Biostar Stem Cell Research Institute, Seoul 08506, Republic of Korea; naraokke@stemcellbio.com (E.Y.K.); kangsk@stemcellbio.com (S.K.K.); 3 Department of Biomedical Laboratory Science, College of Health Science, Eulji University, Uijeongbu 11759, Republic of Korea

**Keywords:** conditioned medium, cumulus–oocyte complex, in vitro maturation, oocyte quality, stem cell, amniotic membrane, PMSG

## Abstract

**Highlights:**

**What are the main findings?**

- Supplementation of IVM medium with 20% hAMSC-CM significantly increased meiotic maturation rates of mouse oocytes (up to 89.4%) across all PMSG priming regimens (0, 5, 10 IU).
- 20% hAMSC-CM combined with 5–10 IU PMSG priming markedly enhanced cleavage and blastocyst formation rates, and upregulated IGF1 and BCL2 in resulting blastocysts.

**What are the implications of the main findings?**

- A dose-optimized, cell-free hAMSC-CM supplement provides a directly transferable, xeno-secretome-based strategy for improving IVM efficiency in assisted reproductive technologies.
- The mechanistic link between AMH/BMP15/GDF9 cumulus signaling, CASP9 suppression in oocytes, and IGF1/BCL2-mediated blastocyst survival offers new molecular targets to refine IVM media for livestock and clinical fertility preservation.

**Abstract:**

In vitro maturation (IVM) of oocytes remains less efficient than in vivo maturation, partly because conventional culture media cannot fully reproduce the follicular microenvironment. This study evaluated whether human amniotic membrane stem cell-conditioned medium (hAMSC-CM) improves IVM efficiency of mouse cumulus–oocyte complexes (COCs) recovered from ICR females primed with 0, 5, or 10 IU pregnant mare serum gonadotropin (PMSG). hAMSC-CM was added at 10% or 20% (*v*/*v*), and maturation, cleavage, blastocyst formation, and gene expression in MII oocytes, cumulus cells, and blastocysts were assessed. Supplementation with 20% hAMSC-CM significantly increased maturation rates across all PMSG groups, reaching 89.4 ± 1.4% versus 59.5 ± 1.3% in the 5 IU PMSG control (*p* < 0.05). With PMSG priming, 20% hAMSC-CM also enhanced cleavage and blastocyst formation. Molecular analyses showed suppression of CASPASE9 in MII oocytes, upregulation of AMH, BMP15, and GDF9 with downregulation of AMHR in cumulus cells, and increased IGF1 and BCL2 expression in blastocysts. These findings indicate that 20% hAMSC-CM, together with optimized PMSG priming, improves mouse oocyte IVM efficiency and embryo developmental competence.

## 1. Introduction

In vitro maturation (IVM) of oocytes is a clinically important technology in both human assisted reproduction and livestock breeding, as it allows immature oocytes retrieved from antral follicles to undergo meiotic resumption and cytoplasmic maturation outside the body [1,2]. Compared with conventional in vitro fertilization (IVF) protocols, IVM significantly reduces the requirement for exogenous gonadotropin stimulation, thereby lowering the risk of ovarian hyperstimulation syndrome and reducing treatment costs [3,4]. Despite these advantages, IVM remains considerably less efficient than in vivo maturation; the rates of meiotic resumption and subsequent embryo development after IVM are generally lower than those achieved following controlled ovarian stimulation [5,6]. This discrepancy is largely attributed to the inability of conventional IVM media to replicate the complex follicular microenvironment, which provides essential paracrine signals, growth factors, and metabolic substrates that support oocyte competence and cytoplasmic maturation [7,8]. Accordingly, supplementation of IVM medium with bioactive molecules that mimic the follicular milieu has been an active area of investigation.

Mesenchymal stem cell-derived conditioned medium (MSC-CM) has emerged as a promising candidate for improving IVM outcomes, given its rich composition of secreted factors including epidermal growth factor (EGF), insulin-like growth factors (IGFs), transforming growth factor-β (TGF-β) family members, vascular endothelial growth factor (VEGF), and numerous cytokines and extracellular vesicles [9,10]. Several studies have reported that supplementation of IVM medium with MSC-CM or MSC-derived extracellular vesicles (EVs) can enhance maturation rates and embryo developmental competence in various species [11,12,13]. Human amniotic membrane-derived stem cells (hAMSCs) are particularly attractive as a source of conditioned medium because they can be obtained from discarded placental tissue without ethical concerns, exhibit low immunogenicity, and secrete an array of trophic factors relevant to reproductive biology [14,15]. Ra et al. [16] previously demonstrated that supplementation of embryo culture medium with 10% hAMSC-CM improved blastocyst formation and hatching rates in mouse IVF, suggesting that biologically active components in hAMSC-CM can positively influence early embryo development. Nevertheless, the optimal concentration of hAMSC-CM for IVM and the molecular mechanisms underlying its beneficial effects on oocyte maturation remain to be elucidated. In addition, although EV-based approaches offer promising avenues for cell-free supplementation, the dose–response relationship and molecular targets in the context of IVM have not been systematically addressed.

Therefore, the objectives of the present study were: (i) to determine the effect of hAMSC-CM supplementation at 10% and 20% (*v*/*v*) on the IVM efficiency, cleavage rate, and blastocyst formation rate of mouse oocytes recovered under different PMSG priming regimens (0, 5, and 10 IU); (ii) to characterize the gene expression profiles of key reproductive and survival-related transcripts—including AMH, AMHR, BMP15, GDF9, IGF1, IGF1R, Klotho, components of the PI3K/AKT/mTOR pathway, and apoptosis-related genes (CASP3, CASP9, BCL2)—in MII oocytes, cumulus cells, and resulting blastocysts; and (iii) to provide mechanistic insights into how hAMSC-CM enhances IVM efficiency by modulating molecular pathways associated with oocyte maturation, follicular signaling, and cell survival.

## 2. Materials and Methods

### 2.1. Animals

Five-week-old female ICR mice were obtained and housed at 24–25 °C with 40–50% relative humidity under a 12 h light/dark cycle. After a 7-day acclimatization period, animals at 6–7 weeks of age were randomly assigned to three groups: (1) control, intraperitoneal (i.p.) injection of 200 µL physiological saline; (2) PMSG 5 IU, i.p. injection of 5 IU PMSG dissolved in 200 µL saline; and (3) PMSG 10 IU, i.p. injection of 10 IU PMSG in 200 µL saline. Mice were euthanized by CO_2_ asphyxiation 46–48 h after injection, and ovaries were surgically removed. Male ICR mice were obtained at the same time at 8 weeks of age and used for sperm collection at 12 weeks of age. All animal experiments were approved by the Institutional Animal Care and Use Committee (IACUC) of Eulji University (approval no. EUIACUC24-16) and conducted in accordance with the relevant guidelines and regulations.

### 2.2. Ovary Collection and Mincing

Following euthanasia, the abdominal skin was disinfected with ethanol and povidone-iodine. The abdominal cavity was opened by cutting through the peritoneum and musculature adjacent to the rib cage using forceps and scissors. Ovaries were carefully excised, avoiding contamination with oviduct and adipose tissue, and placed on sterile gauze to remove blood clots and debris. Each ovary was washed three times in 1 mL physiological saline in a 35-mm Petri dish and transferred to a dish containing 1 mL washing medium [alpha-minimum essential medium (α-MEM) supplemented with 2% polyvinylpyrrolidone (PVP), 5% FBS, and 5% penicillin/streptomycin]. Ovaries were minced under a stereomicroscope using two 30-gauge needles to produce fragments of approximately 1 mm^3^, releasing cumulus–oocyte complexes (COCs) and preantral follicles distributed throughout the cortex [17].

### 2.3. Collection of Immature Oocytes

After mincing, the cell suspension was transferred to a 15-mL conical tube with 2–3 mL washing medium, centrifuged at 300× *g* for 3 min at room temperature, and the pellet was resuspended in fresh washing medium. The suspension was transferred to a 35-mm Petri dish and examined under a stereomicroscope. COCs with an approximate diameter of 120 µm surrounded by 1–2 compact layers of cumulus cells were selected and collected using a mouth pipette [18]. Selected COCs were washed once in washing medium before being transferred to IVM drops.

### 2.4. Preparation and Verification of hAMSC-Conditioned Medium

Human amniotic membrane stem cell-conditioned medium (hAMSC-CM) was provided by Biostar Stem Cell Research Institute (Seoul, Republic of Korea). hAMSC-CM used in this study was prepared using the same method as in our previous report [16], in which its characterization of AMSC and culture methods and CM preparation were described. For the present study, hAMSC-CM was used at final concentrations of 10% and 20% (*v*/*v*) in the IVM base medium.

### 2.5. In Vitro Maturation (IVM)

The IVM base medium consisted of HEPES-buffered α-MEM supplemented with EGF (100 ng/mL; Invitrogen, PHG0314), PMSG (10 IU/mL), and hCG (10 IU/mL). hAMSC-CM was added to the base medium at 10% or 20% (*v*/*v*) to produce the experimental IVM media. A maximum of 20 COCs per 100 µL drop were cultured at 37 °C in 5% CO_2_ for 16–24 h [19]. To prevent potential interference from lipophilic molecules in hAMSC-CM, mineral oil overlay was omitted. Meiotic maturation was confirmed by the presence of the first polar body in the perivitelline space after denudation of cumulus cells by repeated pipetting in 0.6% hyaluronidase (*w*/*v*). Maturation rate was expressed as the percentage of oocytes reaching metaphase II (MII) relative to the total number of COCs cultured.

### 2.6. Sperm Collection and In Vitro Fertilization (IVF)

Male ICR mice (12 weeks) were euthanized by CO_2_ asphyxiation. The scrotum was disinfected and a small incision (~0.5 cm) was made to expose the testes and associated adipose tissue. The cauda epididymis was carefully excised and incised with fine scissors to release the sperm mass. The concentrated sperm mass was dispersed in 200–500 µL CARD medium (Cosmo Bio Co., Tokyo, Japan) and capacitated for 1 h at 36 °C in 5% CO_2_ [20]. Sperm concentration was determined using a Neubauer hemocytometer. MII oocytes from IVM were inseminated in CARD medium at a final concentration of 2 × 10^4^ spermatozoa/100 µL, with up to 20 oocytes per drop, and co-incubated for 3 h at 36 °C [21]. Putative zygotes were washed three times in washing medium and transferred to modified human tubal fluid (mHTF; Cosmo Bio Co.). Embryos were cultured at 37 °C under 5% O_2_ and 5% CO_2_. Cleavage to the 2-cell stage was assessed at 24 h, and blastocyst formation was evaluated at 96 h after fertilization. Cleavage rate (%) was calculated as the number of 2-cell embryos divided by the number of inseminated MII oocytes, and blastocyst rate (%) was calculated as the number of blastocysts divided by the number of cleaved embryos.

### 2.7. Gene Expression Analysis

Total RNA was extracted from pooled MII oocytes, cumulus cells, and resulting blastocysts (minimum of four biological replicates per group) using the easy-spin™ Total RNA Extraction Kit (Intron Biotechnology, Seoul, Republic of Korea). Complementary DNA was synthesized using a reverse transcriptase kit (Intron Biotechnology). Quantitative real-time PCR (qRT-PCR) was performed to assess relative transcript abundance of the following genes: Klotho, IGF1, IGF1R, AMH, AMHR, BMP15, GDF9, PI3K, AKT, MTOR, SMAD1, CASP3, CASP9, and BCL2. Gene expression was normalized to the reference gene β-actin, and relative quantification was performed using the 2^−ΔΔCT^ method. Primer sequences are provided in Table 1.

### 2.8. Statistical Analysis

All experiments were performed with a minimum of seven independent replicates per group for maturation, cleavage, and blastocyst rate assessment, and a minimum of four replicates for gene expression analyses. COCs were randomly allocated to the experimental groups after collection. All treatment groups were processed simultaneously under identical culture conditions to minimize potential confounding effects. Investigators were not blinded during outcome assessment and data analysis. Data were analyzed using Prism 5 software (GraphPad Software, San Diego, CA, USA). One-way analysis of variance (ANOVA) followed by Tukey’s multiple comparison test was used to evaluate differences among groups within each PMSG treatment. Results are expressed as mean ± SEM. Differences were considered statistically significant at *p* < 0.05 and are indicated by different lowercase letters.

## 3. Results

### 3.1. Effect of PMSG Priming on COC Yield and of hAMSC-CM Concentration on IVM Maturation Rate

The number of morphologically intact COCs recoverable by ovarian mincing varied with PMSG dose. No PMSG (0 IU saline control) yielded 10.7 COCs per mouse on average, whereas 5 IU PMSG increased recovery to 14.9 COCs, and 10 IU PMSG yielded 21.5 COCs. Based on these data, PMSG priming at 10 IU was considered optimal for maximizing recovery of competent COCs for IVM (Table S2).

Maturation rates across all PMSG treatment groups are presented in Table 2. In the no-PMSG group, the baseline maturation rate in the control medium was 65.8 ± 4.1% (Table 2). Supplementation with 10% and 20% hAMSC-CM increased maturation rates to 76.6 ± 4.5% and 86.2 ± 3.8%, respectively; the 20% group was significantly higher than the control (*p* < 0.05). In the 5 IU PMSG group, control maturation rate was 59.5 ± 1.3%, increasing to 74.2 ± 0.4% with 10% and 89.4 ± 1.4% with 20% hAMSC-CM (*p* < 0.05). Similarly, in the 10 IU PMSG group, the maturation rate was 59.5 ± 0.7% in the control, 79.7 ± 1.1% with 10%, and 88.8 ± 2.4% with 20% hAMSC-CM (*p* < 0.05). Across all PMSG priming conditions, supplementation with 20% hAMSC-CM consistently yielded significantly higher maturation rates compared with the respective control groups (*p* < 0.05), identifying 20% as the optimal supplementation concentration. In 10 IU PMSG-treated oocytes, 30% and 50% hAMSC-CM yielded significantly higher IVM efficiency than 20% (Table S1). Subsequent gene expression analyses were therefore conducted using oocytes and embryos derived from the PMSG 10 IU priming protocol. 

Cleavage and blastocyst formation rates of MII oocytes after IVF were also strongly influenced by the PMSG priming dose, and these dose-dependent differences were markedly more pronounced than those observed for the maturation rate alone (Table 2, Table S2). In the no-PMSG (0 IU) group, cleavage rates remained low across all hAMSC-CM conditions (43.88 ± 3.30% in the control, 40.37 ± 1.87% with 10%, and 44.91 ± 2.13% with 20% hAMSC-CM), and blastocyst formation was almost negligible (0 ± 0%, 1.43 ± 1.43%, and 11.52 ± 1.69%, respectively), indicating that hAMSC-CM alone, in the absence of gonadotropin priming, was insufficient to support efficient developmental competence even when meiotic maturation reached 86.2%. In contrast, gonadotropin-primed groups exhibited a substantial gain in both endpoints: in the 5 IU PMSG group, cleavage rates increased to 66.19 ± 2.35% (control), 76.27 ± 2.20% (10%), and 74.38 ± 5.37% (20% hAMSC-CM), and blastocyst formation rates reached 30.37 ± 7.36% in the 5 IU/20% hAMSC-CM group, which was significantly higher than the 0 IU/0% control (*p* < 0.05). The most pronounced developmental improvement was observed under 10 IU PMSG priming, where cleavage rates were significantly higher in the 10% (83.69 ± 2.33%) and 20% (84.17 ± 4.59%) hAMSC-CM groups than in the no-PMSG controls (*p* < 0.05), and blastocyst formation rates rose to 19.44 ± 2.70% (10%) and 28.34 ± 4.19% (20%). Importantly, although the maturation-enhancing effect of 20% hAMSC-CM was evident across all PMSG conditions, the conversion of MII oocytes into developmentally competent embryos was clearly dependent on PMSG priming, with 5 and 10 IU PMSG producing approximately a 2- to 3-fold and 19- to 30-fold increase, respectively, in blastocyst yield relative to the corresponding no-PMSG conditions. These results indicate that, while 20% hAMSC-CM is the most effective supplement for nuclear maturation, optimal gonadotropin priming (5–10 IU PMSG) is essential to translate the improved maturation into superior cleavage and blastocyst development.

### 3.2. Gene Expression in MII Oocytes

**Figure 1:**
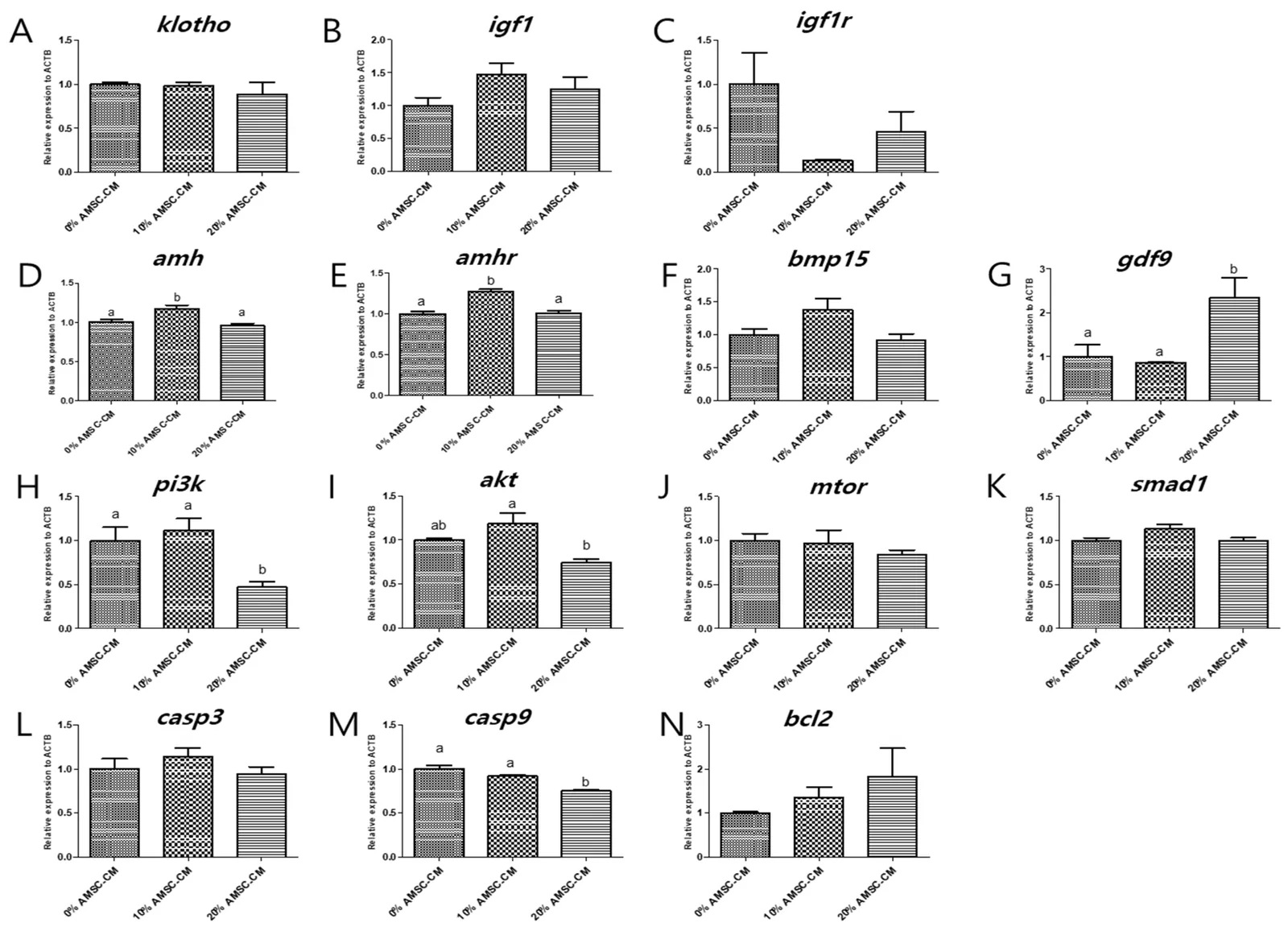
Relative gene expression in matured oocytes supplemented with 0% (control), 10%, or 20% hAMSC-CM during IVM. Transcripts analyzed: (**A**) klotho, (**B**) igf1, (**C**) igf1r, (**D**) amh, (**E**) amhr, (**F**) bmp15, (**G**) gdf9, (**H**) pi3k, (**I**) akt, (**J**) mtor, (**K**) smad1, (**L**) casp3, (**M**) casp9, (**N**) bcl2. Data represent mean ± SEM (n ≥ 4 replicates). Different lowercase letters indicate significant differences (*p* < 0.05; one-way ANOVA with Tukey’s post hoc test).

Relative transcript levels in MII oocytes are shown in Figure 1. Klotho, IGF1, and IGF1R transcripts did not differ significantly among the three groups. SMAD1 transcript levels also did not differ significantly among the three groups. AMH transcript abundance in 10% hAMSC-CM-treated oocytes was higher than in control oocytes (*p* < 0.05), while AMHR expression was higher in the 10% group compared with control. The 20% hAMSC-CM group did not differ significantly from control for either AMH or AMHR transcripts. Among oocyte maturation-related growth factors, BMP15 expression did not differ significantly across groups; however, GDF9 transcript levels were higher in the 20% hAMSC-CM group compared with control (*p* < 0.05). Regarding the PI3K/AKT/MTOR signaling pathway, PI3K transcript abundance in the 20% hAMSC-CM group was significantly lower than in both the control and the 10% group (*p* < 0.05). AKT transcripts were also significantly reduced in the 20% group compared with the 10% group (*p* < 0.05), whereas MTOR expression did not differ significantly among groups, indicating that the PI3K/AKT reduction did not propagate to the downstream MTOR level. For apoptosis-related transcripts, CASP9 was significantly lower in the 20% hAMSC-CM group compared with control and the 10% group (*p* < 0.05), whereas CASP3 and BCL2 did not differ significantly among groups.

### 3.3. Gene Expression in Matured Cumulus Cells

**Figure 2:**
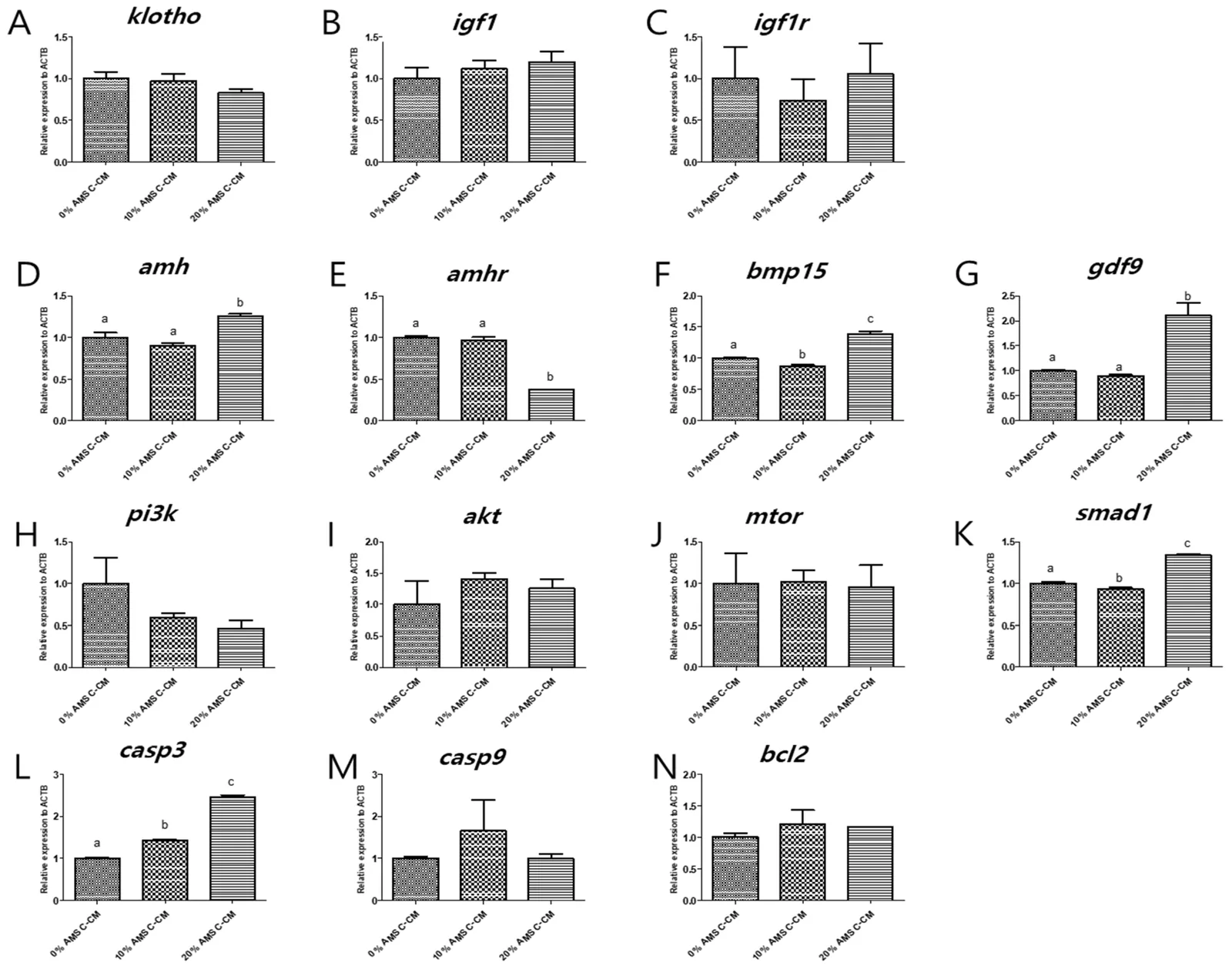
Relative gene expression in matured cumulus cells supplemented with 0% (control), 10%, or 20% hAMSC-CM during IVM. Transcripts analyzed: (**A**) klotho, (**B**) igf1, (**C**) igf1r, (**D**) amh, (**E**) amhr, (**F**) bmp15, (**G**) gdf9, (**H**) pi3k, (**I**) akt, (**J**) mtor, (**K**) smad1, (**L**) casp3, (**M**) casp9, (**N**) bcl2. Data represent mean ± SEM (n ≥ 4 replicates). Different lowercase letters indicate significant differences (*p* < 0.05; one-way ANOVA with Tukey’s post hoc test).

Gene expression data from cumulus cells are presented in Figure 2. Klotho, IGF1, and IGF1R transcript levels did not differ significantly among groups. SMAD1 transcript levels also did not differ significantly among groups. AMH transcript abundance was significantly elevated in the 20% hAMSC-CM group compared with both control and the 10% group (*p* < 0.05). Conversely, AMHR transcripts were significantly downregulated in the 20% group relative to control and the 10% group (*p* < 0.05), suggesting a negative-feedback response to elevated AMH signaling. BMP15 and GDF9 expression were both significantly higher in the 20% group than in control and the 10% group (*p* < 0.05). PI3K transcript levels showed a declining trend with increasing hAMSC-CM concentration, but differences were not statistically significant. AKT and MTOR expression also did not differ significantly among groups. For apoptosis-related markers, CASP3 was paradoxically elevated in the 20% group relative to control (*p* < 0.05) and the 10% group (*p* < 0.05). CASP9 and BCL2 expression did not differ significantly among groups.

### 3.4. Gene Expression in Blastocysts Derived from IVM Oocytes

**Figure 3:**
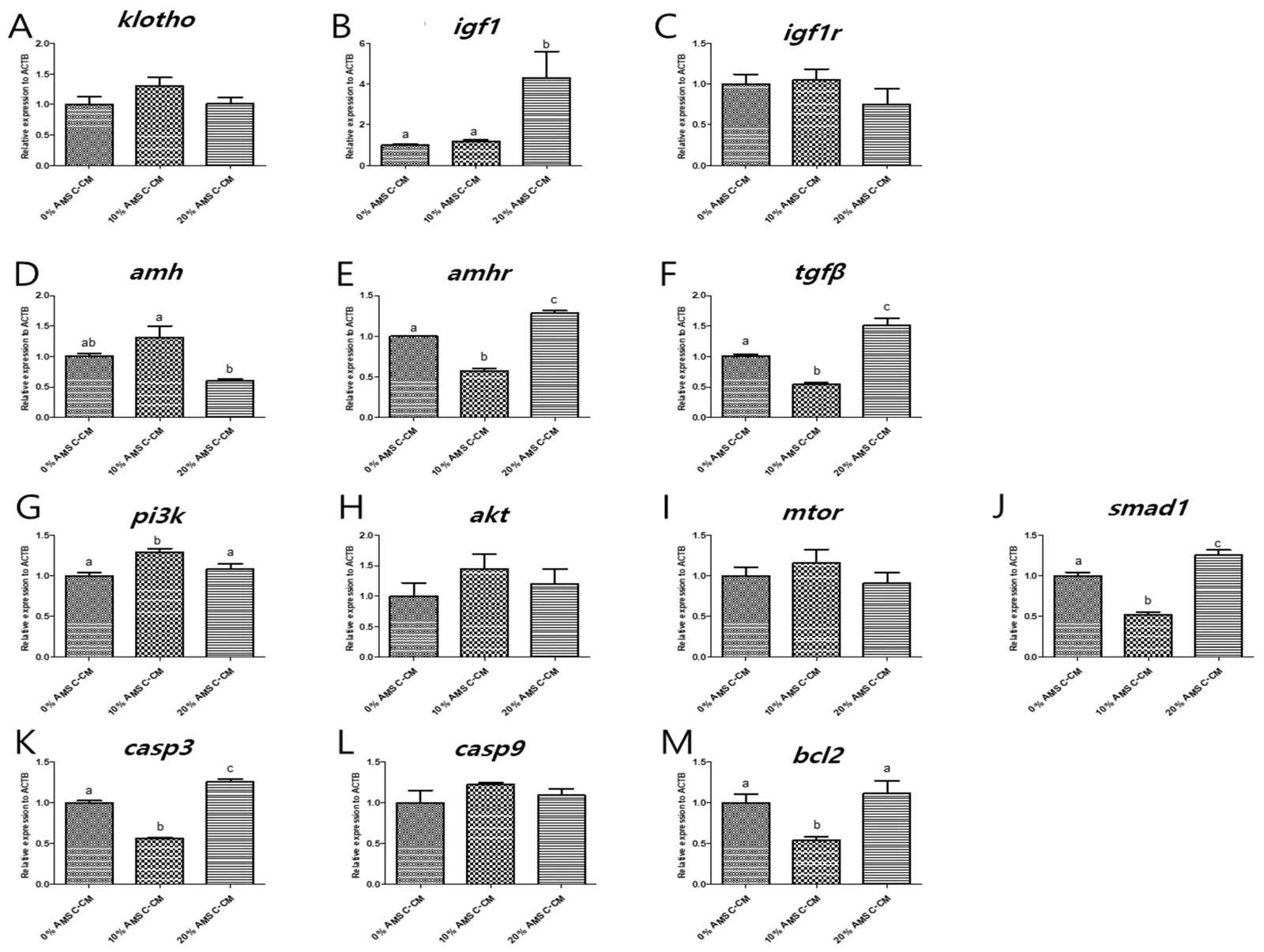
Relative gene expression in blastocysts derived from oocytes matured in vitro with 0% (control), 10%, or 20% hAMSC-CM. Transcripts analyzed: (**A**) klotho, (**B**) igf1, (**C**) igf1r, (**D**) amh, (**E**) amhr, (**F**) tgfβ, (**G**) pi3k, (**H**) akt, (**I**) mtor, (**J**) smad1, (**K**) casp3, (**L**) casp9, (**M**) bcl2. Data represent mean ± SEM (n ≥ 4 replicates). Different lowercase letters indicate significant differences (*p* < 0.05; one-way ANOVA with Tukey’s post hoc test).

Transcript profiles in blastocysts are shown in Figure 3. Klotho and IGF1R did not differ significantly among groups. IGF1 expression was dramatically upregulated in the 20% hAMSC-CM group than control and the 10% group (*p* < 0.05). AMH expression was significantly higher in the 10% group than in the 20% group; neither differed significantly from control. AMHR and TGFβ expression showed the opposite pattern: both were highest in the 20% group (*p* < 0.05), whereas the 10% group expressed these transcripts at lower levels than control. Similarly, SMAD1 was highest in the 20% group (*p* < 0.05). PI3K transcript was highest in the 10% group (*p* < 0.05); AKT and MTOR did not differ among groups. CASP3 was highest in the 20% group (*p* < 0.05) and lowest in the 10% group. BCL2 expression was significantly elevated in the control and the 20% hAMSC-CM groups relative to the 10% group (*p* < 0.05).

## 4. Discussion

The present study demonstrates that supplementation of IVM medium with 20% hAMSC-CM significantly improves the meiotic maturation rate of mouse oocytes across different PMSG priming regimens, achieving maturation rates of up to 89.4% compared with approximately 60% in control medium. These findings are consistent with the previous reports that MSC-derived secretomes can enhance the follicular microenvironment and support oocyte competence [9,11,12]. The improvement observed with hAMSC-CM is likely attributable to the combined actions of multiple growth factors, cytokines, and EVs present in the conditioned medium, which may collectively recapitulate aspects of the paracrine signaling network ordinarily provided by granulosa and somatic cells within the antral follicle [14,15]. The dose-dependent effect, with 20% outperforming 10%, suggests that a threshold concentration of bioactive factors is required to exert maximal stimulatory effects on meiotic resumption and cytoplasmic maturation.

The reduction in CASP9 transcript levels in MII oocytes treated with 20% hAMSC-CM is noteworthy. CASP9 is indispensable for the elimination of oocytes with unrepaired double-strand DNA breaks during prophase I of meiosis [22]. Its downregulation in the 20% group therefore implies that fewer oocytes experienced irreparable DNA damage during IVM, reflecting improved chromosomal integrity and overall oocyte quality. This interpretation is supported by pharmacological studies in which specific CASP9 inhibitors reduced cellular stress and apoptosis in embryos, leading to improved developmental outcomes [23]. Concomitantly, the elevation of GDF9 transcripts in 20% hAMSC-CM-treated MII oocytes is consistent with a role for GDF9 in promoting meiotic resumption and cytoplasmic maturation through autocrine and paracrine mechanisms [24]. Together, these molecular changes in MII oocytes indicate that 20% hAMSC-CM supports higher-quality meiotic maturation compared with the control or 10% conditions.

In cumulus cells, the upregulation of AMH, BMP15, and GDF9 transcripts by 20% hAMSC-CM highlights the importance of cumulus–oocyte bidirectional communication in mediating the beneficial effects of the conditioned medium. AMH is expressed predominantly in cumulus and mural granulosa cells of growing follicles and is associated with follicle quality; elevated AMH in cumulus cells of dominant follicles has been observed in both bovine and human models [25,26]. AMH supplementation during IVM or IVF has been shown to improve maturation and blastocyst development rates in mice and humans [27,28], and treatment of female mice with AMH enhances oocyte quality and litter size [29]. The concomitant downregulation of AMHR observed in cumulus cells of the 20% group is consistent with a negative-feedback response to elevated AMH signaling, a mechanism that may modulate the extent of AMH-mediated follicular activation. The coordinated upregulation of BMP15 and GDF9—both critical oocyte-derived factors known to drive cumulus expansion, granulosa cell differentiation, and oocyte competence [30]—further supports the notion that 20% hAMSC-CM creates a signaling milieu in cumulus cells that is favorable for IVM. The paradoxical elevation of CASP3 transcripts in cumulus cells of the 20% group warrants careful interpretation. Cumulus cell apoptosis during IVM is considered inversely related to oocyte developmental competence [31]; however, CASP3 can also participate in non-lethal signaling functions, and its mRNA upregulation does not necessarily equate to active caspase-3 protein cleavage or execution of cell death. The absence of significant changes in CASP9 and BCL2 transcripts in cumulus cells suggests that the intrinsic mitochondrial apoptotic pathway was not uniformly activated. Bidirectional gap-junction communication between cumulus cells and the oocyte is critical for cytoplasmic maturation [32]; thus, the net impact of altered CASP3 expression on oocyte quality in this context requires further protein-level investigation.

The most compelling evidence for the beneficial effect of 20% hAMSC-CM supplementation during IVM came from the gene expression profile of resulting blastocysts. The pronounced upregulation of IGF1 in blastocysts from the 20% group is particularly significant, as IGF-related gene expression has been proposed as a molecular marker of embryo quality and developmental potential [33]. The simultaneous elevation of BCL2, a potent anti-apoptotic protein that participates in the mitochondria-dependent survival pathway, reinforces the interpretation that resulting blastocysts from the 20% group possessed superior cell viability. Growth factors from the maternal environment drive preimplantation embryo proliferation and differentiation through receptor-mediated signaling cascades, including the PI3K pathway activated by IGF1 and its receptor [34,35,36,37,38]. The elevated TGFβ and SMAD1 transcripts in blastocysts from the 20% group are consistent with activation of BMP/SMAD signaling, which is known to regulate cell lineage specification in the blastocyst [39]. Although CASP3 was also elevated in blastocysts from the 20% group, this was counterbalanced by proportionally higher BCL2 expression, suggesting net cell survival rather than apoptotic execution, as previously noted for PI3K-modulated embryo survival [38]. The relatively lower PI3K expression in the 20% group compared with the 10% group, paradoxically associated with higher embryo quality markers, may reflect context-dependent regulatory interactions that merit further investigation at the protein level.

Notably, the increased SMAD1 expression observed in blastocysts from the 20% hAMSC-CM group may reflect reinforced BMP-responsive signaling during preimplantation development rather than a nonspecific transcriptional change. Nuclear pSMAD1 activity has been demonstrated in early mouse blastocysts, and BMP signaling is required for proper trophectoderm and primitive endoderm development; conversely, BMP inhibition increases cleaved CASP3-positive cells in preimplantation embryos [39]. In this context, the concurrent elevation of TGFβ and BCL2 in the 20% group suggests that enhanced SMAD1 signaling may be linked to a more favorable developmental and pro-survival state. Because fragmented blastocysts tend to exhibit lower BCL2 levels [40], the present SMAD1-high/BCL2-high profile is more consistent with improved embryo quality than with activation of apoptotic execution. Nevertheless, additional protein-level analyses of pSMAD1 and lineage markers will be needed to clarify whether this response primarily reflects strengthened extra-embryonic lineage specification, survival signaling, or both.

Regarding PMSG priming, the present results confirm that PMSG at 10 IU provides a roughly 2-fold greater yield of morphologically competent COCs compared with unprimed controls, while 5 IU provides an intermediate benefit. Importantly, the dose of PMSG strongly influenced not only COC recovery but also the subsequent cleavage and blastocyst formation rates: cleavage rates in the no-PMSG group did not exceed 45% irrespective of hAMSC-CM concentration, whereas priming with 5 or 10 IU PMSG raised cleavage to 66–84% and lifted blastocyst rates from near zero to 28–30% under 20% hAMSC-CM supplementation. These results indicate that an appropriate level of gonadotropin priming is essential for translating improved meiotic maturation into developmentally competent embryos. In this regard, recent evidence by Lin et al. [41] indicates that excessive doses of equine chorionic gonadotropin (eCG; 15 IU), although increasing the absolute number of oocytes recovered, significantly reduced fertilization, four-cell, and blastocyst formation rates in mouse IVF (e.g., blastocyst rate of 44.68% at 15 IU vs. 74.72% at 0 IU), and elevated oxidative stress, mitochondrial dysfunction, and chromosomal aneuploidy in the resulting embryos. Although the present study examined IVM-derived oocytes rather than fully in vivo-matured ones, the PMSG dosages used here (5 and 10 IU) fall within the lower, non-toxic range described by Lin et al. [41], which may explain why both cleavage and blastocyst rates were preserved or even enhanced when combined with 20% hAMSC-CM. Together, these comparisons suggest that there exists a therapeutic window for PMSG/eCG priming, in which 5–10 IU optimally balances COC yield with embryo developmental competence, whereas higher doses are likely to provoke oxidative stress-related damage that conditioned medium supplementation cannot fully compensate for. The maturation-enhancing effect of 20% hAMSC-CM was evident across all three PMSG regimens, indicating that its beneficial effects on nuclear maturation are not dependent on a specific gonadotropin-priming background; however, gonadotropin dose remains a key determinant of downstream developmental success.

Recent evidence further supports the beneficial role of human amniotic membrane-derived stem cell secretomes in reproductive biotechnology. La et al. [42] reported that extracellular vesicles (EVs) derived from human amniotic mesenchymal stem cells significantly improved oocyte quality and embryonic development in aged mice through enhanced antioxidant capacity, mitochondrial function, and activation of the Keap1/Nrf2 signaling pathway. These findings are broadly consistent with the present study, in which hAMSC-CM supplementation improved oocyte maturation and subsequent embryo development. However, several important distinctions should be emphasized. First, La et al. utilized purified EVs and quantified treatment doses based on particle concentration, whereas the present study evaluated whole conditioned medium supplemented at volumetric concentrations (10% and 20%, *v*/*v*), thereby reflecting a more practically applicable IVM supplementation strategy. Second, the previous study focused primarily on rescuing age-associated declines in oocyte quality, whereas our study investigated enhancement of developmental competence during routine IVM conditions. Third, while La et al. primarily examined oxidative stress and mitochondrial regulatory mechanisms, the present study additionally evaluated maturation rates, cleavage and blastocyst formation outcomes, and gene expression profiles in mature oocytes, cumulus cells, and resulting blastocysts. Taken together, these studies suggest that multiple components within the hAMSC secretome, including extracellular vesicles and soluble paracrine factors, may contribute to improved oocyte competence and embryo development through complementary biological mechanisms.

Several limitations of the present study merit acknowledgment. First, although hAMSC-CM used in this study was prepared using the same method as in our previous report [16], the present work did not include comprehensive batch-specific characterization of its bioactive components. Recent evidence suggests that extracellular vesicles derived from hAMSCs exert beneficial effects on oocyte quality through antioxidant and mitochondrial regulatory mechanisms [42]. Because conditioned medium contains both EVs and numerous soluble bioactive factors, the relative contribution of each component to the observed improvement in oocyte maturation and embryo development remains unclear. Therefore, future studies involving proteomic profiling, EV isolation, particle quantification, and comparative functional analyses of whole conditioned medium versus purified EV fractions will be necessary to identify the principal mediators responsible for the observed biological effects. Second, the present study assessed only transcript-level changes using qRT-PCR; therefore, the proposed molecular mechanisms should be interpreted with appropriate caution. Alterations in gene expression do not necessarily correspond to changes in protein abundance or signaling activity because post-transcriptional and post-translational regulatory mechanisms may influence biological responses. Accordingly, the pathways discussed herein should be regarded as potential mechanisms associated with hAMSC-CM supplementation rather than definitive evidence of pathway activation or inhibition. Future studies should validate both the expression and activation of key signaling molecules, including CASP3, CASP9, BCL2, AKT, mTOR, SMAD1, and IGF1, using protein-level approaches such as Western blotting, immunofluorescence, or immunohistochemistry. Such analyses will be essential to confirm the biological significance of the transcriptional changes observed in the present study. Third, embryo developmental competence was evaluated primarily by cleavage and blastocyst formation rates together with transcriptional analyses. Although these developmental endpoints are widely accepted indicators of preimplantation development, they do not fully reflect overall embryo quality. Future studies incorporating total blastocyst cell counts, differential inner cell mass/trophectoderm (ICM/TE) staining, apoptosis assays (e.g., TUNEL), and embryo transfer experiments will be necessary to comprehensively evaluate embryo quality and implantation potential following hAMSC-CM supplementation. Third, embryo developmental competence was evaluated primarily by cleavage and blastocyst formation rates together with transcriptional analyses. Although these developmental endpoints are widely accepted indicators of preimplantation development, they do not fully reflect overall embryo quality. Future studies incorporating total blastocyst cell counts, differential inner cell mass/trophectoderm (ICM/TE) staining, apoptosis assays (e.g., TUNEL), and embryo transfer experiments will be necessary to comprehensively evaluate embryo quality and implantation potential following hAMSC-CM supplementation. Fourth, translation of the present findings to other species—particularly livestock or human oocytes—awaits systematic investigation. Notwithstanding these limitations, the present study provides a mechanistic framework suggesting that hAMSC-CM enhances mouse oocyte IVM through coordinated modulation of AMH/BMP15/GDF9 signaling in cumulus cells, suppression of meiotic DNA-damage checkpoints (CASP9) in oocytes, and promotion of IGF1- and BCL2-mediated survival in resulting blastocysts. Importantly, because the murine COC-IVM system serves as the foundational translational model for assisted reproductive technologies (ART) in domestic species, the dose-optimized, cell-free hAMSC-CM supplementation protocol established here offers a directly transferable framework for refining IVM media in livestock (cattle, swine, small ruminants) and companion animals (equine, canine), where suboptimal IVM efficiency remains a critical bottleneck for embryo production, genetic improvement, and fertility preservation. Future cross-species validation studies in bovine and porcine COCs are therefore warranted to confirm the broader applicability of hAMSC-CM as a standardized, xeno-secretome-based supplement for animal reproductive biotechnology.

## 5. Conclusions

In summary, the present study demonstrates that supplementing IVM medium with 20% hAMSC-CM is an effective and reproducible strategy for enhancing mouse oocyte meiotic maturation and subsequent embryonic developmental competence. The maturation-enhancing effect of 20% hAMSC-CM was consistent across 0, 5, and 10 IU PMSG priming conditions, achieving maturation rates of up to 89.4%. However, the translation of this improved maturation into superior cleavage and blastocyst formation required appropriate gonadotropin priming (5–10 IU PMSG). The observed transcriptional changes may be associated with coordinated upregulation of *AMH*/*BMP15*/*GDF9* signaling in cumulus cells, suppression of the meiotic DNA-damage checkpoint gene *CASP9* in oocytes, and elevated *IGF1* and *BCL2* expression in resulting blastocysts. Together, these findings establish a dose-optimized, cell-free hAMSC-CM supplementation protocol that may provide a useful experimental framework for future studies aimed at refining IVM media in livestock and companion animals.

## Figures and Tables

**Table 1:** Primer sequences used for qRT-PCR.

Gene	Description	NCBI Accession No.	Forward Primer (5′→3′)	Reverse Primer (5′→3′)	Product Size (bp)
*Klotho*	alpha-klotho (Kl), transcript variant X1, mRNA	NM_013823	cccggaagtctttgctggt	atgtgtggaatgcggctca	201
*IGF1*	insulin-like growth factor 1, transcript variant 1	NM_010512.5	gctggtggatgctcttcagt	tagggacggggacttctgag	244
*IGF1R*	insulin-like growth factor I receptor	NM_010513.3	gtgtggatcgcgatttctgc	gtacatgctctgggtgctgt	134
*AMH*	anti-Müllerian hormone	NM_007445.3	aacctttgtgcctggtgaca	ttctccagtctcccctagcc	224
*AMHR*	anti-Müllerian hormone type 2 receptor	NM_001356575.1	taccagctgctaggcctaca	atgagcacattctggctgct	284
*BMP15*	bone morphogenetic protein 15	NM_009757.5	tgtggggagtggtgcttttt	tgcttcatctccttgccagg	142
*GDF9*	growth differentiation factor 9	NM_001439459.1	ctgacaggtctggcctcttg	gccggacggtattgtagagg	180
*PI3K*	phosphoinositide-3-kinase regulatory subunit 1	NM_001024955.2	gaggatttgccccaccatga	ccactacggagcaggcatag	148
*AKT*	thymoma viral proto-oncogene 1	NM_001331107.2	aatgtgggctcatgggtctg	agagggagagggccagttag	152
*mTOR*	mechanistic target of rapamycin kinase	NM_020009.2	aaggcctgatgggatttggg	ggggcagcaggttaaggatt	174
*SMAD1*	SMAD family member 1	NM_008539.4	agtgggctttcatcaggctc	gcaaagagcatatgcctgcc	206
*CASP3*	caspase 3	NM_001284409.1	gtcatctcgctctggtacgg	cacacacacaaagctgctcc	169
*CASP9*	caspase 9, transcript variant 1	NM_015733.6	tggctcctggtacatcgaga	ccccgagcctcatgaagttt	184
*BCL2*	B cell lymphoma 2	NM_009741.5	tacgagtgggatgctggaga	cggtagcgacgagagaagt	236
*TGFβ*	transforming growth factor, beta 1	NM_011577.2	ccgcaacaacgccatctatg	tgccgtacaactccagtgac	273
*ACTB*	actin, beta	NM_007393.5	ccaccatgtacccaggcatt	actcctgcttgctgatccac	177

**Table 2:** In vitro maturation (IVM), cleavage, and blastocyst formation rates of mouse oocytes supplemented with 0%, 10%, or 20% hAMSC-CM under 0, 5, and 10 IU PMSG priming conditions. Data are mean ± SEM (n ≥ 7 replicates per group). Different superscript letters within a column indicate significant differences (*p* < 0.05; one-way ANOVA with Tukey’s post hoc test).

PMSG Dose (IU)	hAMSC-CM (%)	Maturation Rate (%)	Cleavage Rate (%)	Blastocyst Rate (%)
0	0	65.8 ± 4.1 ^a^	43.88 ± 3.30 ^a^	0 ± 0 ^a^
0	10	76.6 ± 4.5 ^ab^	40.37 ± 1.87 ^a^	1.43 ± 1.43 ^a^
0	20	86.2 ± 3.8 ^b^	44.91 ± 2.13 ^ab^	11.52 ± 1.69 ^ab^
5	0	59.5 ± 1.3 ^a^	66.19 ± 2.35 ^ab^	11.63 ± 0.92 ^ab^
5	10	74.2 ± 0.4 ^ab^	76.27 ± 2.20 ^ab^	14.65 ± 3.31 ^ab^
5	20	89.4 ± 1.4 ^b^	74.38 ± 5.37 ^ab^	30.37 ± 7.36 ^b^
10	0	59.5 ± 0.7 ^a^	77.65 ± 2.86 ^ab^	13.26 ± 2.43 ^ab^
10	10	79.7 ± 1.1 ^ab^	83.69 ± 2.33 ^b^	19.44 ± 2.70 ^ab^
10	20	88.8 ± 2.4 ^b^	84.17 ± 4.59 ^b^	28.34 ± 4.19 ^b^

## Data Availability

The original contributions presented in this study are included in the article/Supplementary Material. Further inquiries can be directed to the corresponding authors.

## Supplementary Materials

The following supporting information can be downloaded at: https://www.mdpi.com/article/10.3390/cells15161454/s1, Table S1: Effect of hAMSC-CM Concentration on In Vitro Maturation Rate of Mouse Oocytes; Table S2: No. animals, collected COCs (cumulus cells-oocytes complexes), matured cleaved embryos, and blastocysts supplemented with 0%, 10%, or 20% hAMSC-CM under 0, 5, and 10 IU PMSG priming conditions.

## Abbreviations

The following abbreviations are used in this manuscript:

AMH	Anti-Müllerian Hormone
AMHR	Anti-Müllerian Hormone Receptor
ART	Assisted Reproductive Technologies
BCL2	B-cell lymphoma 2
BMP15	Bone Morphogenetic Protein 15
CASP3	Caspase 3
CASP9	Caspase 9
COC	Cumulus–Oocyte Complex
EGF	Epidermal Growth Factor
EV	Extracellular Vesicle
eCG	Equine Chorionic Gonadotropin
GDF9	Growth Differentiation Factor 9
hAMSC-CM	Human Amniotic Membrane Stem Cell-Conditioned Medium
hCG	Human Chorionic Gonadotropin
IACUC	Institutional Animal Care and Use Committee
IGF1	Insulin-like Growth Factor 1
IGF1R	Insulin-like Growth Factor 1 Receptor
IVF	In Vitro Fertilization
IVM	In Vitro Maturation
MII	Metaphase II
MSC-CM	Mesenchymal Stem Cell-Conditioned Medium
mTOR	Mechanistic Target of Rapamycin
PI3K	Phosphoinositide-3-kinase
PMSG	Pregnant Mare Serum Gonadotropin
qRT-PCR	Quantitative Real-Time Polymerase Chain Reaction
SEM	Standard Error of the Mean
SMAD1	SMAD Family Member 1
TGFβ	Transforming Growth Factor Beta
VEGF	Vascular Endothelial Growth Factor
α-MEM	Alpha-Minimum Essential Medium
