# Supplementary material for: Efficacy of Human Amniotic Membrane Stem Cell-Conditioned Medium on Improving In Vitro Maturation Efficiency of Mouse Oocytes
Source: Cells. 2026 Aug 13;15(16):1454. doi: 10.3390/cells15161454 (PMC13511258; doi:10.3390/cells15161454)
Supplement: Supplementary file 1 [file cells-15-01454-s001.zip › cells-4397876-supplementary.pdf]

Supplementary Materials

Title: Efficacy of Human Amniotic Membrane Stem Cell-Conditioned Medium on Improving In Vitro Maturation Efficiency of Mouse Oocytes

Table S1. Effect of hAMSC-CM Concentration on In Vitro Maturation Rate of Mouse Oocytes

**hAMSC-CM Concentration (%)**	**Total Oocytes** No.	**Immature Oocytes** No.	**Matured Oocytes** No.	**IVM Rate (%) (Mean ± SEM)**
**0**	354	218	136	61.88 ± 1.68b
**20**	372	200	172	85.61 ± 0.90a
**30**	334	202	132	66.33 ± 3.19b
**50**	329	208	121	58.73 ± 1.63b

Values are expressed as mean ± SEM of 9 independent biological replicates per group. In each replicate, all immature oocytes (GV stage) collected were subjected to IVM in medium supplemented with the indicated concentration of hAMSC-CM for 18–24 h. Matured oocytes were defined as those extruding the first polar body (MII stage). IVM rate (%) was calculated as (matured oocytes / total oocytes) × 100 for each replicate, and the mean ± SEM was computed across replicates. Statistical significance was assessed by one-way ANOVA followed by Tukey's HSD post-hoc test. Different superscript letters (a, b) indicate statistically significant differences among groups ($p < 0.05$). The 20% hAMSC-CM group showed a significantly higher IVM rate compared to all other groups ($p < 0.001$), whereas the 0%, 30%, and 50% groups did not differ significantly from each other.

Table S2. No. animals, collected COCs (cumulus cells-oocytes complexes), matured cleaved embryos, and blastocysts supplemented with 0%, 10%, or 20% hAMSC-CM under 0, 5, and 10 IU PMSG priming conditions.

**PMSG dose (IU)**	**hAMSC-CM (%)**	**No. Animals**	**No. Collected COCs**	**No. Matured oocytes**	**No. Cleaved embryos**	**No. Blastocyst**
0	0	18	68	43	19	0
0	10		79	58	23	1
0	20		62	53	24	6
5	0	11	60	35	23	4
5	10		57	42	32	6
5	20		43	38	28	11
10	0	11	66	39	30	5
10	10		60	48	40	9
10	20		60	55	46	15
